# Adherence of the General Public to Self-Protection Guidelines During the COVID-19 Pandemic

**DOI:** 10.1017/dmp.2020.445

**Published:** 2020-11-18

**Authors:** Parnian Jabbari, Nazanin Taraghikhah, Forouq Jabbari, Saied Ebrahimi, Nima Rezaei

**Affiliations:** 1 Research Center for Immunodeficiencies, Children’s Medical Center, Tehran University of Medical Sciences, Tehran, Iran; 2 Network of Immunity in Infection, Malignancy and Autoimmunity (NIIMA), Universal Scientific Education and Research Network (USERN), Tehran, Iran; 3 Maternal, Fetal and Neonatal Research Center, Tehran University of Medical Sciences, Tehran, Iran; 4 Research Department of Rajaei Heart Center, Iran University of Medical Sciences, Tehran, Iran; 5 Department of Immunology, School of Medicine, Tehran University of Medical Sciences, Tehran, Iran

**Keywords:** COVID-19, pandemic, personal protective equipment, self-protection guideline, transmissible diseases

## Abstract

**Objective:**

The coronavirus disease (COVID-19) pandemic is rapidly growing due to a high level of contagiousness. Different measures have been taken to slow the spread of the virus. Appropriate use of personal protective equipment (PPE) is one of these key measures. In this cross-sectional study, we investigated adherence of the general public to use of PPE and their knowledge regarding the rationale behind their use.

**Methods:**

Two samples were chosen from public places (a subway station and a city store) in Tehran, Iran, one of the countries affected by COVID-19. Individuals were observed for appropriate use of PPE and interviewed regarding their knowledge on some basic self-protection information.

**Results:**

Approximately half of the 431 participants did not take any measures to ensure hand hygiene, whereas those who did not use respiratory protection were far fewer. A considerable number of individuals, however, did not use these PPE correctly. On the other hand, there was a gap in the knowledge of the general public regarding different aspects of protective measures. The majority of the participants were receptive toward education on preventive measurements through public media.

**Conclusion:**

Education is an important aspect in containing the COVID-19 pandemic, as it directly increases adherence of the general public to protective measures.

## Introduction

An epidemic of severe acute respiratory syndrome coronavirus 2 (SARS-CoV-2) started in Hubei Province of China, late 2019, which became a pandemic in a matter of weeks.^1^ Shortly, official authorities enforced stay-at-home and other physical-distancing orders, following guidelines provided by valid public health authorities in order to decelerate person-to-person transmission of the virus (www.cdc.gov, www.who.int). These guidelines covered an expanding list of clinical manifestations, routes of transmission, and rational use of personal protective equipment (PPE). The guidelines regarding use of PPE were modified shortly due to the shortage of such equipment in the face of the rapidly growing pandemic. These interim guidelines divided use of PPE based on risk of exposure and recommended that the general public use cloth face coverings in order to spare highly specialized PPE such as N95 respirators and surgical masks for health care providers. These guidelines also designated features of effective cloth face coverings.

In response to the PPE shortages, many organizations engaged in providing these alternative PPE by manufacturing cloth face coverings and hand sanitizers. However, it is not clear to what extent these PPE conform to the features designated by the aforementioned guidelines. Therefore, it is important that the general public has a fair knowledge regarding the effectiveness of these PPE in order to ensure cessation of transmission.

As the restrictions are being decreased in country after country, cessation of person-to-person transmission depends highly on adherence of the general public to the official guidelines. This calls for an estimation of knowledge of the general public of such guidelines. Herein, we investigated adherence of the general public to, and indirectly their knowledge of, these guidelines in Tehran, Iran, one of the first countries severely affected by the virus.

## Methods and Materials

The current study was an analytical, cross-sectional study designed to investigate adherence of the Iranian general public to guidelines designated to decelerate person-to-person transmission of the virus. The sample populations were aimed to represent individuals who engage in social activities where physical-distancing was not feasible and according to the health guidelines use of PPE was highly recommended/mandatory. Therefore, individuals running errands in one of the City Stores of Tehran city, as well as individuals in one of the populated subway stations of this city on a Tuesday morning (workday) in June were chosen. Data were collected in 1 day of all individuals embarking the subway (between 8:00 AM and 9:00 AM) and entering the City Store (between 10:00 AM and 11:00 AM) who were observed for use of PPE and interviewed for knowledge on use of PPE and physical-distancing measures. This research was exempt by our university’s institutional review board.

Respiratory PPE was divided into 2 categories of cloth face covering as compared to N95 respirators and surgical masks. Hand-sanitation equipment included use of plastic/cotton gloves as well as hand sanitizers. Knowledge of personal protection measures was evaluated by interrogating recommended physical-distancing as well as features of standard hand sanitizer. In an additional evaluation, individuals were asked of their preferred platform to be educated regarding self-protective measures, which included public media (television, posters, and newspapers), online platforms (social media), and person-to-person education (telephone calls and campaigns). A scoring system was developed in order to compare use of PPE among the 2 sample populations: for respiratory protection, use of surgical mask, N95 respirators, or 2-layered cotton mask covering nose and mouth and secured under the chin: 2 points; non-conformation of mask to standard features or incorrect use of face covering: 1 point; use of face shield alone: 1 point; no respiratory protection through nose/mouth covering: 0 points; for hand hygiene, use of both hand sanitizers and gloves: 2 points; use of hand sanitizer or gloves: 1 point; no measures of hand sanitation: 0 points. Knowledge of individuals regarding self-protection guidelines was assessed based on the following scoring system: reporting a distance of > 6 feet (or 1.5 meters) for physical-distancing: 2 points; reporting a distance of < 6 feet (or 1.5 meters): 1 point; statement of no knowledge regarding physical-distancing guidelines: 0 points; describing a minimum of 60% for alcohol content of hand sanitizers: 2 points; describing alcohol contents below 60%: 1 point; statement of no knowledge regarding the alcohol content of standard hand sanitizers: 0 points.

A 2-sample t-test was used to evaluate whether the mean scores for use of protective respiratory (score 0 = no face covering, score 1 = incorrect use of face covering, and score 2 = appropriate face covering) and hand-sanitation equipment (score zero = no measure, score 1 = glove OR hand sanitizer, score 2 = glove AND hand sanitizer) were different between the 2 groups. Analyses were performed in R 4.0.0.

## Results

A total of 431 individuals (163 persons running errands and 268 subway passengers, 175 females and 256 males) were included in this study. For use of respiratory PPE, the majority (78.35%) of subway passengers wore face coverings, irrespective of their category, whereas 57.46% used hand-sanitation equipment either in the form of gloves or hand sanitizers. Of those running errands, only 46.01% wore face covering, and use of hand sanitation was limited to 44.78% of individuals (Table 1).

**Table 1.** Summary of the study interviews and observations

Subway (N = 268)			
Hand hygiene	Gloves	N = 73	
	Disinfectant	N = 119	
Face covering	Cloths	Correct	N = 0
		Incorrect	N = 67
	Surgical	Correct	N = 91
		Incorrect	N = 52
	Shields	Only	N = 2
		+ mask	N = 9
City Store (N = 163)			
Hand hygiene	Gloves	N = 45	
	Disinfectant	N = 44	
Face covering	Cloths	Correct	N = 0
		Incorrect	N = 25
	Surgical	Correct	N = 25
		Incorrect	N = 25
	Shields	Only	N = 3
		+ mask	N = 1
General Awareness (N = 431)			
Social distancing	No awareness	N = 28	
	Incorrect	N = 26	
	Correct	N = 377	
Standard disinfectant	No awareness	N = 197	
	Incorrect	N = 66	
	Correct	N = 163	
Preferred education platform	Public media	N = 265	
	Online platforms	N = 111	
	Person-to-person	N = 48	
	No preference	N = 7	

Among 193 individuals using surgical masks or N95 respirators, 60.1% used their masks correctly at the moment of observation. A total of 226 individuals used hand-sanitation equipment: 46.01% individuals used only hand sanitizers, and 26.1% used only gloves for hand sanitation.

**Figure 1.**
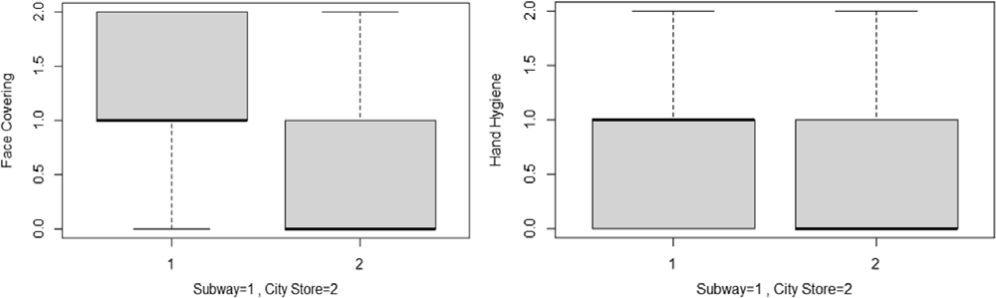
Use of PPE between 2 study samples. Face covering: score 0 = no PPE; score 1 = incorrect use; score 2 = appropriate use. Hand hygiene: score 0 = no measure; score 1 = glove **or** hand sanitizer; score 2 = glove **and** hand sanitizer.

We compared use of protective measures among subway passengers and individuals running errands. Use of face covering (*P* value = 1.905e^-11^; 95% CI: 0.36–0.65) and hand-sanitation equipment (*P* value = 0.002; 95% CI: 0.07–0.35) among subway passengers was significantly higher than those running errands (Figure 1).

Table 1 summarizes other aspects of the interview, including popular educative platform and knowledge of participants on self-protection measures/equipment.

## Discussion

The path of any outbreak can be determined by its reproductive number (R_0_). An R_0_ > 1 indicates continued spread of the infection, whereas R_0_ ≤ 1 leads to vanishment of the infection. For the case of SARS-CoV-2, the average R_0_ is estimated to be 3.3.^2^ This high rate of spread coupled with the lack of a vaccine^3^ and definite treatment^4^ makes the future of this pandemic dependent on other preventive measures.^5^ The stay-at-home orders seem to have played an important role in containing the spread^2^; however, due to different challenges posed by these orders, it was not feasible to continue them. This is why we need to ensure adherence to public health guidelines. Our study mainly aimed to gain an estimation of this adherence.

We noted that, even though individuals are willing to use PPE, they might not be aware of the rationale behind the use of PPE.^6^ None of the cloth face coverings in our study conformed to the features provided by the guidelines. This is alarming as having a face covering may lead to false reassurance, thus, minimizing the physical distance. On the other hand, a considerable proportion (≈ 40%) of those who used surgical face masks or N95 respirators, which are mostly available in black markets in critical times such as pandemics, did not use these PPE correctly and this number might even be higher, as the observation time of our study was short. This can translate to wasting valuable, highly effective PPE which can be used in more appropriate settings until their shortage is resolved. Furthermore, many individuals relied on the use of gloves to maintain hygiene of hands, even though the guidelines emphasized that wearing gloves will not necessarily guarantee appropriate hand hygiene. Altogether, these facts lead to the assumption that individuals are not well familiar with the guidelines, rationale use of PPE, and mechanism of spread of virus, even though the latter is still under investigation. We also believe that appropriate rules and continuous education can be a solution to slow the spread of the virus, as has been the case with the spread of other viral diseases creating pandemics in the past, such as the HIV/AIDS pandemic.^7^

Our assumptions are further backed up by the results of our study. The comparison of the 2 sample populations regarding use of PPE shows significantly better hand hygiene and respiratory protection among subway passengers. A couple of factors may be the reason behind this significant difference between the 2 sample populations extracted from the same greater population, that is, citizens of Tehran city: the current rules for use of the subway, which mandates use of PPE for all subway passengers along with frequent visual reminders, such as placards. However, this cannot be the only reason, as there are no penalties for those who violate these rules, as seen in some instances in our study. Another factor can be presumptions of the one’s ability to maintain physical distance. Those running errands may assume that they can maintain a safe distance from other individuals, while subway passengers may be aware that physical distancing is not feasible in the subway. This can be another example of unawareness of guidelines as use of PPE is also recommended for running errands.

We further hypothesized that education can be a key factor in eliminating the spread of the virus.^7^ This was supported by the awareness of the individuals regarding the recommended physical distance, which has been emphasized through different platforms. On the other hand, our reports are not indicative of such level of awareness on features of standard hand sanitizers, as these features have been less discussed. This calls for constant education and emphasis on adherence to guidelines through appropriate platforms.

Even though this study provides information on adherence of the general public to self-protection guidelines, there are some limitations to this study. In order to avoid recall bias, individuals were not asked about their use of PPE in public places before the COVID-19 pandemic. Another limitation was lack of formal education regarding preventive measures. As individuals could not be divided into groups who had received formal education and those who had not, drawing conclusions directly indicating effectiveness of education based on statistical data was not possible.

It would provide interesting information if similar studies were performed in other cities of Iran, as well as other countries. However, performing a comparison between the adherence of individuals to self-protection guidelines in different cities and countries requires information on spread of COVID-19 in each city and country, the former not readily available in many countries, including Iran.

## Conclusion

As individuals are back to having a social life, authorities must make sure of their abiding to guidelines. The most appropriate and feasible means to this goal are the constant education to increase awareness of the general public regarding the spread of the virus and ways to protect one’s self from infection.
